# Supplementary material for: Identification of a cross-neutralizing antibody that targets the receptor binding site of H1N1 and H5N1 influenza viruses
Source: Nat Commun. 2022 Sep 2;13:5182. doi: 10.1038/s41467-022-32926-5 (PMC9439264; doi:10.1038/s41467-022-32926-5)
Supplement: Supplementary file 1 — Supplementary Information [file 41467_2022_32926_MOESM1_ESM.pdf]

Supplementary Information

Identification of an Antibody with Cross-neutralization of H1N1 and H5N1 Influenza Viruses that Target Receptor Binding Site

Tingting Li[1,2,#], Junyu Chen[1,2,#], Qingbing Zheng[1,2,#], Wenhui Xue[1,2,#], Limin Zhang[1,2,#], Rui Rong[1,2], Sibo Zhang[1,2], Qian Wang[1,2], Minqing Hong[1,2], Yuyun Zhang[1,2], Lingyan Cui[1,2], Maozhou He[1,2], Zhen Lu[1,2], Zhenyong Zhang[1,2], Xin Chi[1,2], Jinjin Li[1,2], Yang Huang[1,2], Hong Wang[1,2], Jixian Tang[1,2], Dong Ying[1,2], Lizhi Zhou[1,2], Yingbin Wang[1,2], Hai Yu[1,2], Jun Zhang[1,2], Ying Gu[1,2,*], Yixin Chen[1,2,*], Shaowei Li[1,2,*], Ningshao Xia[1,2,3,*]

[#] These authors contributed equally to this work.

[*] Correspondence should be addressed to N.X.(nsxia@xmu.edu.cn), S.L.(shaowei@xmu.edu.cn), Y.C.(yxchen2008@xmu.edu.cn)

Y.G.(guying@xmu.edu.cn)

This PDF file includes:

Supplementary Figures 1 to 16
Supplementary Tables 1 to 9

Supplementary Figures

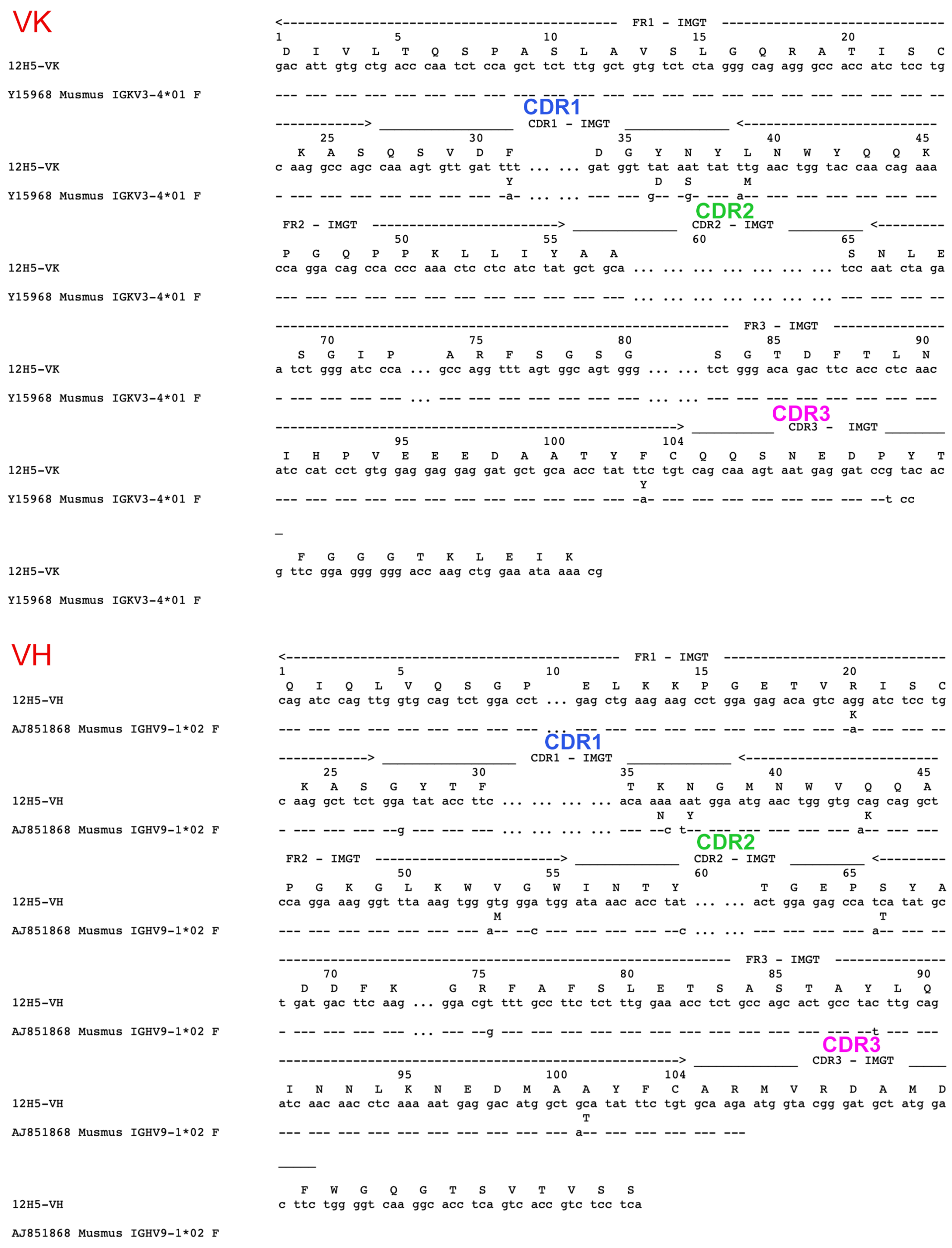

Supplementary Fig. 1. Nucleotide and amino acid sequences of the VL and VH chain regions of C12H5. DNA sequences of the VK and VH regions of C12H5 were compared with the closest germline sequences and analysed using the IMGT database (http://www.imgt.org). Complementarity-determining regions (CDRs) are indicated in blue, green, and purple font for CDRs 1, 2, and 3, respectively.

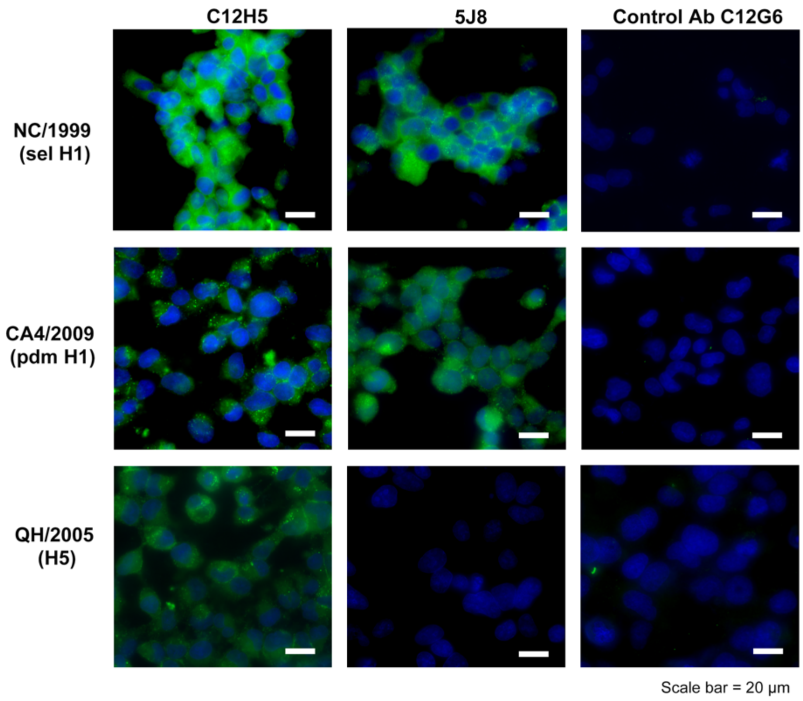

Supplementary Fig. 2. Immunofluorescence assay activity for C12H5 against three strains of influenza A viruses. MDCK cells infected with either A/New Caledonia/20/1999 (Seasonal H1N1, NC/1999), A/California/04/2009 (Pandemic H1N1, CA4/2009), or A/Bar-headed Goose/Qinghai/15C/2005 (H5N1, QH/2005) were treated with C12G6, control antibody (5J8) or negative control antibody (C12G6) and stained green. Data were presented from one experiment.

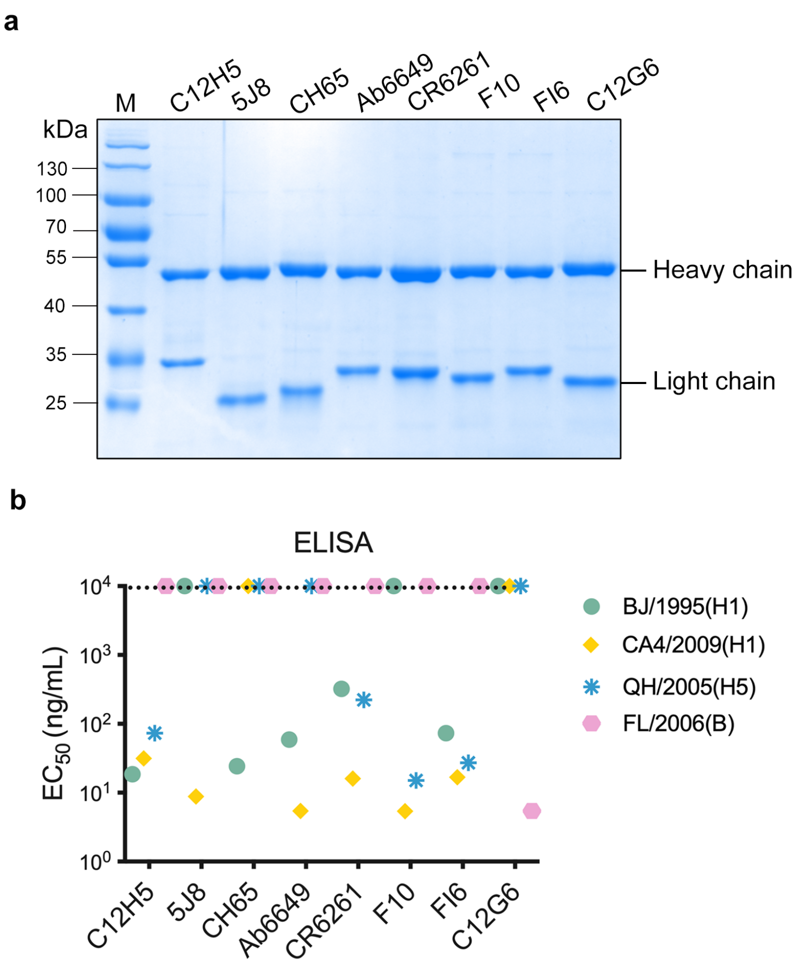

Supplementary Fig. 3. Expression, purification, and characterization of influenza A HA bnAbs. a SDS-PAGE analysis of purified C12H5, 5J8, CH65, Ab6649, CR6261, F10, FI6, and C12G6 antibodies. Data were presented from one representative experiment. **b** Characterization of the activities of C12H5, 5J8, CH65, Ab6649, CR6261, F10, FI6, and C12G6 antibodies using ELISA. Shown are the EC_{50} values for binding of the antibodies to purified HA of various influenza A virus strains. EC_{50} values above 10^4 ng/ml (dotted line) were scored as unfavourable. Source data are provided as a Source Data file.

	Strains	MN-IC$_{50}$(μg/mL)							
		C12H5	5J8	CH65	Ab6649	CR6261	F10	FI6	C12G6
sel H1N1	TX/1991	0.07	2.31	0.07	7.98	20.12	2.88	13.26	>50
	HK/1994	0.23	0.17	0.14	11.28	>50	8.84	26.01	>50
	BJ/1995	0.15	>50	0.59	>50	>50	14.73	35.36	>50
	NC/1999	0.08	>50	2.96	10.67	35.36	7.98	17.17	>50
	SI/2006	6.50	>50	3.56	9.20	25.00	4.60	9.38	>50
	BR/2007	8.84	>50	6.50	9.45	15.95	1.78	2.52	>50
pdm H1N1	CA4/2009	2.30	0.07	>50	18.39	29.47	10.06	14.73	>50
	CA7/2009	4.42	0.34	>50	15.95	17.68	8.84	15.95	>50
	XM/2009	4.42	0.07	>50	>50	39.81	7.11	15.29	>50
	HK/2010	15.95	0.14	>50	25.00	35.36	25.00	13.01	>50
	HK/2011	17.68	2.92	>50	12.50	14.23	7.11	7.11	>50
	XM/2015	9.20	0.08	>50	>50	5.03	1.30	3.01	>50
H5N1	QH/2005	4.20	>50	>50	>50	31.91	7.98	4.30	>50
	XJ/2006	6.50	>50	>50	>50	5.89	7.11	11.32	>50
B	FL/2006	>50	>50	>50	>50	>50	>50	>50	0.11

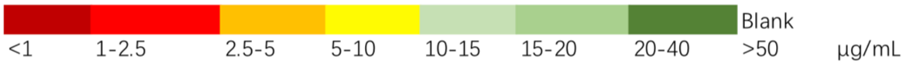

Supplementary Fig. 4. In vitro neutralization activities (IC$_{50}$ values) of C12H5 and other reported influenza A HA bnAbs. Fifty percent inhibitory concentrations (IC$_{50}$) of C12H5, 5J8, CH65, Ab6649, CR6261, F10, and FI6 antibodies and the control antibody, C12G6, against representative strains from the three influenza A lineages and an influenza B virus were determined by performing microneutralization (MN) assays. The values are representative of three independent experiments and are reported as mean values. The values below 50 μg/ml are colour-filled; dark red, extreme reactivity; red, strong reactivity; orange, moderate-high reactivity; yellow, moderate-low reactivity; light green, weak reactivity; green, very weak reactivity; >50, negative reactivity.

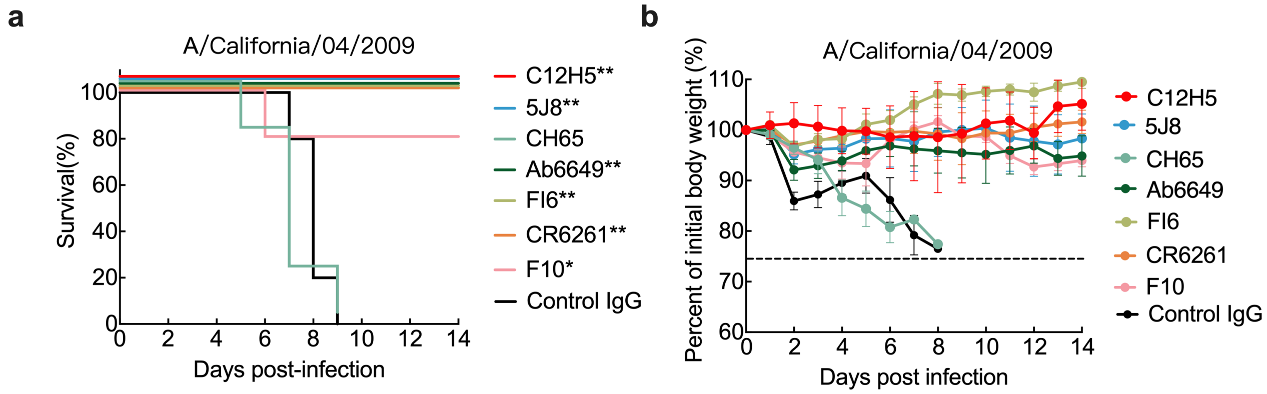

Supplementary Fig. 5. Comparison of therapeutic efficacies of C12H5 and other bnAbs in mice. Survival curves (**a**) and body weight changes (**b**) for BALB/c mice (n = 5 per group) treated intravenously with antibodies (15 mg/kg for MA-CA4/2009 virus) 24 h after lethal challenge with 25 MLD50 of MA-CA4/2009 virus. Data in **b** are presented as mean values ± SD. Statistical analyses in **a** were performed using the log-rank test. $^{*}P < 0.05$ and $^{**}P < 0.01$ compared with the control IgG-treated group. Source data are provided as a Source Data file.

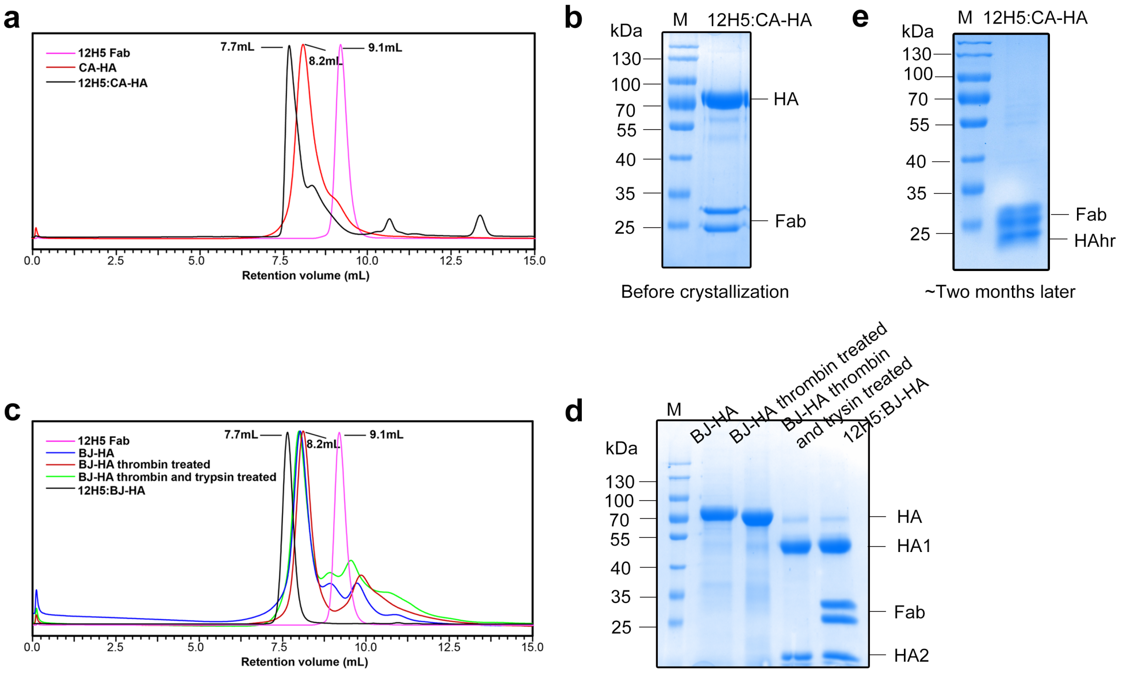

Supplementary Fig. 6. Complex preparation of 12H5: HA^{CA} and 12H5: HA^{BJ} immune complexes. a HPLC profiles of HA trimer without protease treatment and its complex with 12H5 Fab. Due to the instability of protease treated-CA HA, trimer CA HA with foldon was prepared for immune complex. **b** SDS-PAGE analysis of purified 12H5: HA^{CA} complex. **c** Protease treatment of BJ HA. Before protease treatment, BJ HA was resolved as a full-length HA0 and separate HA1 and HA2 in SDS-PAGE. The foldon facilitating HA trimerization was removed with thrombin, and the thrombin-treated sample was further catalysed with trypsin, demonstrating that HA0 was completely cleaved to HA1 and HA2. **d** The purified 12H5: HA^{BJ} immune complex sample was further analysed by SDS-PAGE. **e** SDS-PAGE analysis of the complex of 12H5:CA-HA before crystallization and after storage for two months, the HA are degraded into one ~20 kDa band that is corresponded to the HA head domain in the crystal lattice. Data in **b, d, e** were presented from one representative experiment. Source data are provided as a Source Data file.

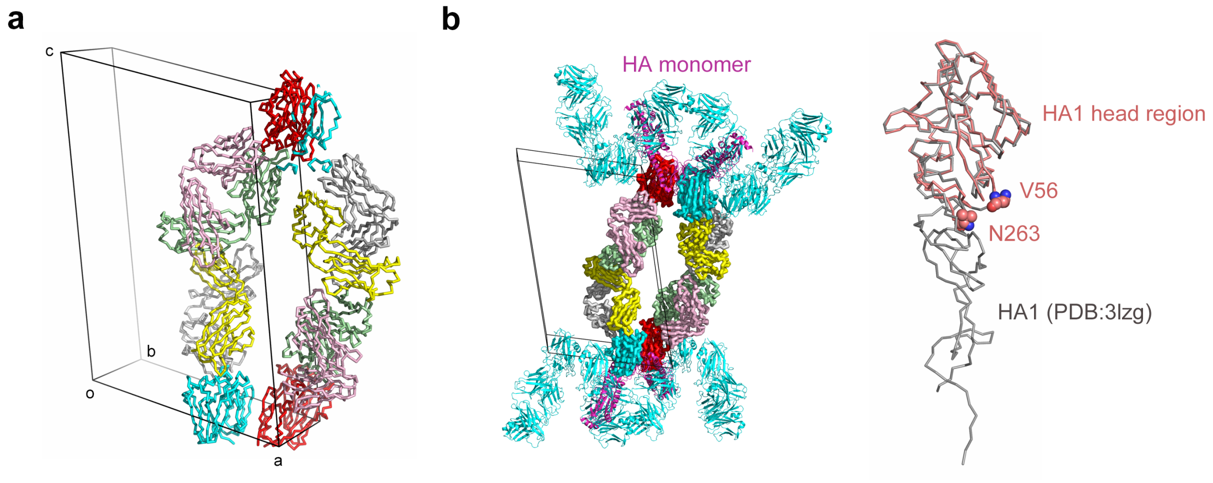

c

```
                     <11                                                        56                                    83a
CA09 HA region      ---------- ---------- ---------- ---------- ------VAPL HLGKCNIAGW ILGNPECESL STASSWSYIV
CA09 HA             DTLCIGYHAN NSTDTVDTVL EKNVTVTHSV NLLEDKHNGK LCKLRGVAPL HLGKCNIAGW ILGNPECESL STASSWSYIV
                      90                                        125a       133a                  150
CA09 HA region      ETPSSDNGTC YPGDFIDYEE LREQLSSVSS FERFEIFPKT SSWPNHDSNK GVTAACPHAG AKSFYKNLIW LVKKGNSYPK
CA09 HA             ETPSSDNGTC YPGDFIDYEE LREQLSSVSS FERFEIFPKT SSWPNHDSNK GVTAACPHAG AKSFYKNLIW LVKKGNSYPK
                                                                                        230
CA09 HA region      LSKSYINDKG KEVLVLWGIH HPSTSADQQS LYQNADTYVF VGSSRYSKKF KPEIAIRPKV RDQEGRMNYY WTLVEPGDKI
CA09 HA             LSKSYINDKG KEVLVLWGIH HPSTSADQQS LYQNADTYVF VGSSRYSKKF KPEIAIRPKV RDQEGRMNYY WTLVEPGDKI
                                         263
CA09 HA region      TFEATGNLVV PRYAFAMERN ---------- ---------- ---------- ---------- ---------- ----------
CA09 HA             TFEATGNLVV PRYAFAMERN AGSGIIISDT PVHDCNTTCQ TPKGAINTSL PFQNIHPITI GKCPKYVKST KLRLATGLRN
                       329><1
CA09 HA region      ---------- ---------- ---------- ---------- ---------- ---------- ---------- ----------
CA09 HA             IPSIQSRGLF GAIAGFIEGG WTGMVDGWYG YHHQNEQGSG YAADLKSTQN AIDEITNKVN SVIEKMNTQF TAVGKEFNHL

CA09 HA region      ---------- ---------- ---------- ---------- ---------- ---------- ---------- ----------
CA09 HA             EKRIENLNKK VDDGFLDIWT YNAELLVLLE NERTLDYHDS NVKNLYEKVR SQLKNNAKEI GNGCFEFYHK CDNTCMESVK
                                         174>
CA09 HA region      ---------- ---------- -
CA09 HA             NGTYDYPKYS EEAKLNREEI D
```

Supplementary Fig. 7. Crystal structure information of 12H5: HAhr^{CA} complex. a Crystal packing of the 12H5: HAhr^{CA} complex. The crystal has a P_{21} space group. There are two 12H5: HAhr^{CA} complexes in one crystallographic asymmetric unit. **b** Superimposition of the HA head region on the HA (left) or HA1 (right) monomer in the reported crystal structure (PDB code: 3lzg). **c** Sequence alignment of the CA HA head region (aa 56 to 263; H3 numbering) resolved in the 12H5: HAhr^{CA} crystal and the HA construct (consisting of HA1 [aa 11 to 329] and HA2 [aa 1 to 174]). The dashed red lines denote the corresponding residues that are missing in the crystal structure.

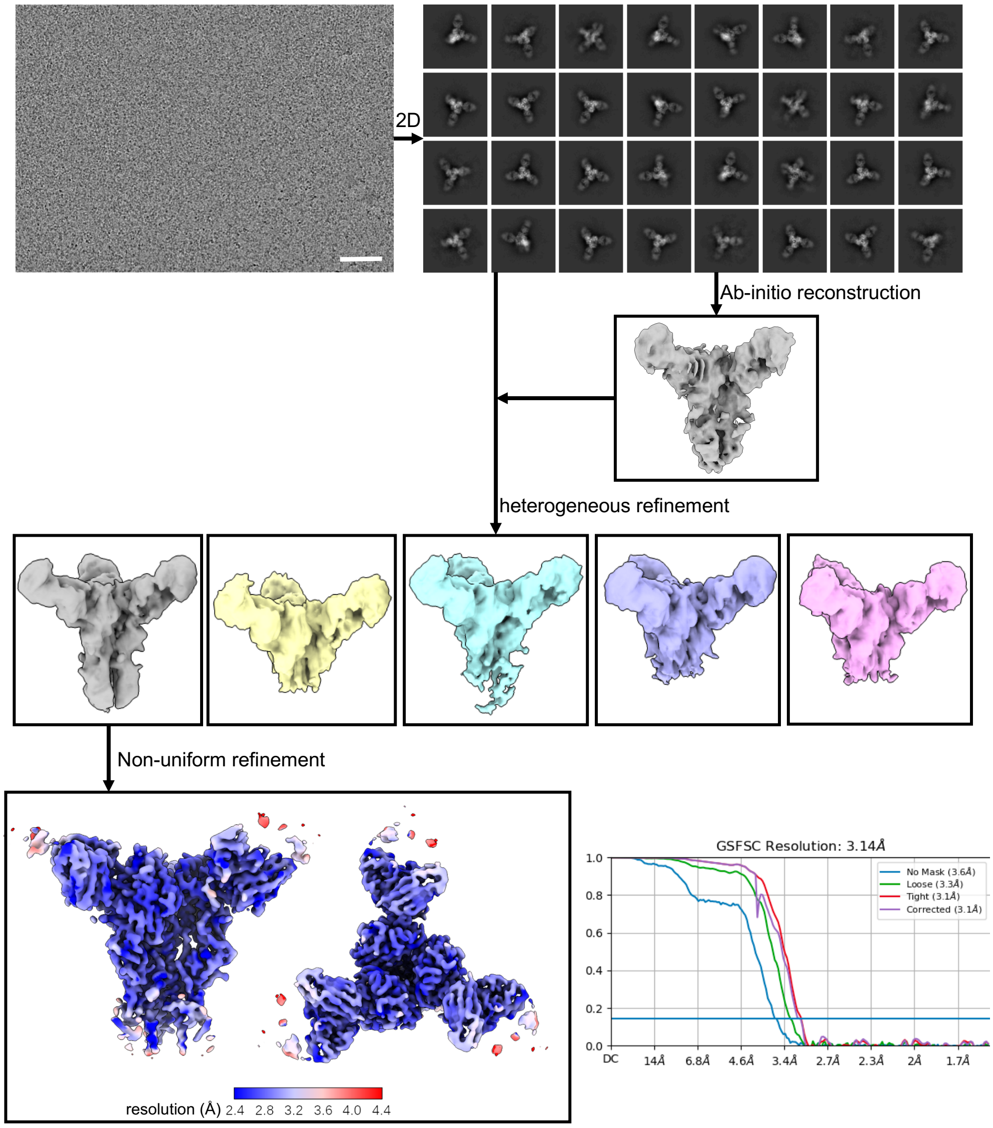

Supplementary Fig. 8. Single-particle cryo-EM image processing workflow for the immune complex of 12H5: HA^{BJ}. Representative electron micrograph, 2D classifications, ab-initio reconstruction, heterogeneous refinement, non-uniform refinement and local refinement maps are shown. Scale bar: 50 nm.

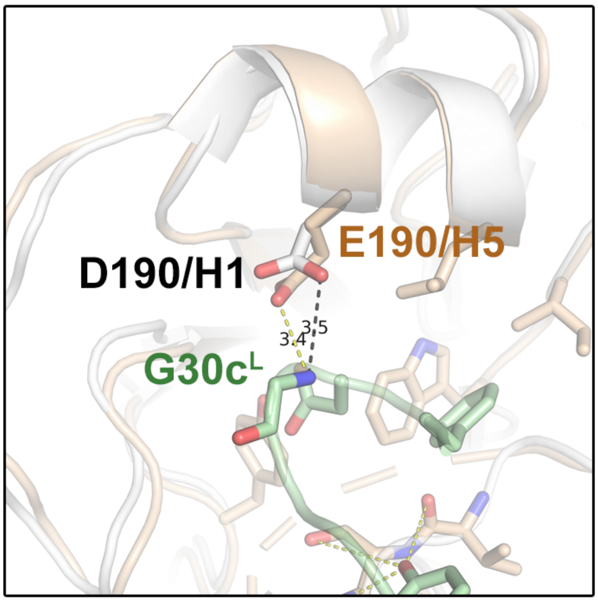

Supplementary Fig. 9. Close-up view of the side chains of D and E at the 190 position of HA and the hydrogen bonds that form with the main chain of C12H5 G30c^{L}.12H5: HAhr^{CA} complex is aligning to the H5 structure (PDB code: 3UBE). Hydrogen bond between G30c^{L} and D190 is shown as black dashed lines. The probable hydrogen bond between G30c^{L} and E190 is shown as yellow dashed lines.

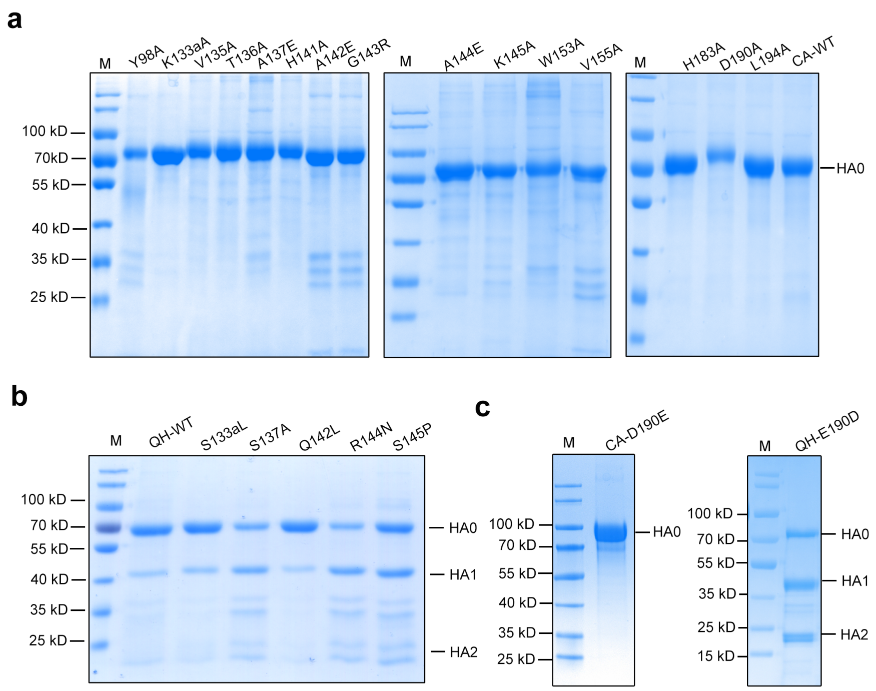

Supplementary Fig. 10. Characterization of HA mutants with structure-based alanine substitutions at 12H5 binding sites. a SDS-PAGE analysis of the purified CA4/2009 HA mutants. **b** SDS-PAGE analysis of the purified QH/2005 HA mutants. **c** SDS-PAGE analysis of the purified CA4/2009 D190E and QH/2005 E190D HA mutants. Data in **a, b, c** were presented from one representative experiment. Source data are provided as a Source Data file.

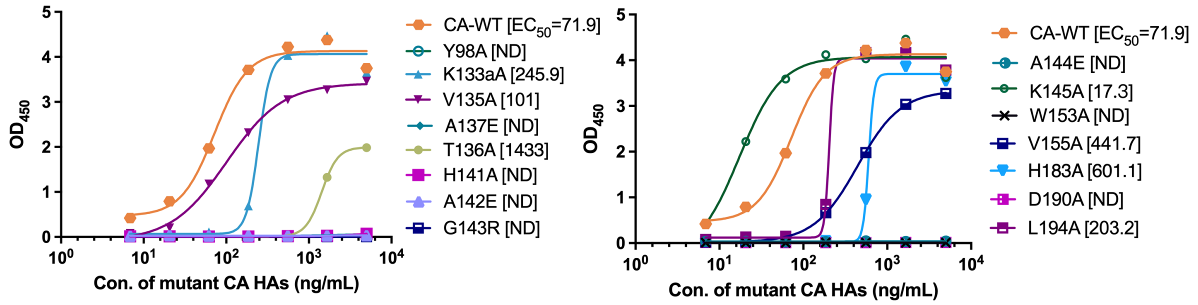

Supplementary Fig. 11. Double-antibody sandwich ELISA for the interaction of HA and mutants against MAb C12H5. EC50s were calculated from two experiments by sigmoid trend fitting and are plotted as in Fig. 6d. OD_{450}, OD at 450 nm. Source data are provided as a Source Data file.

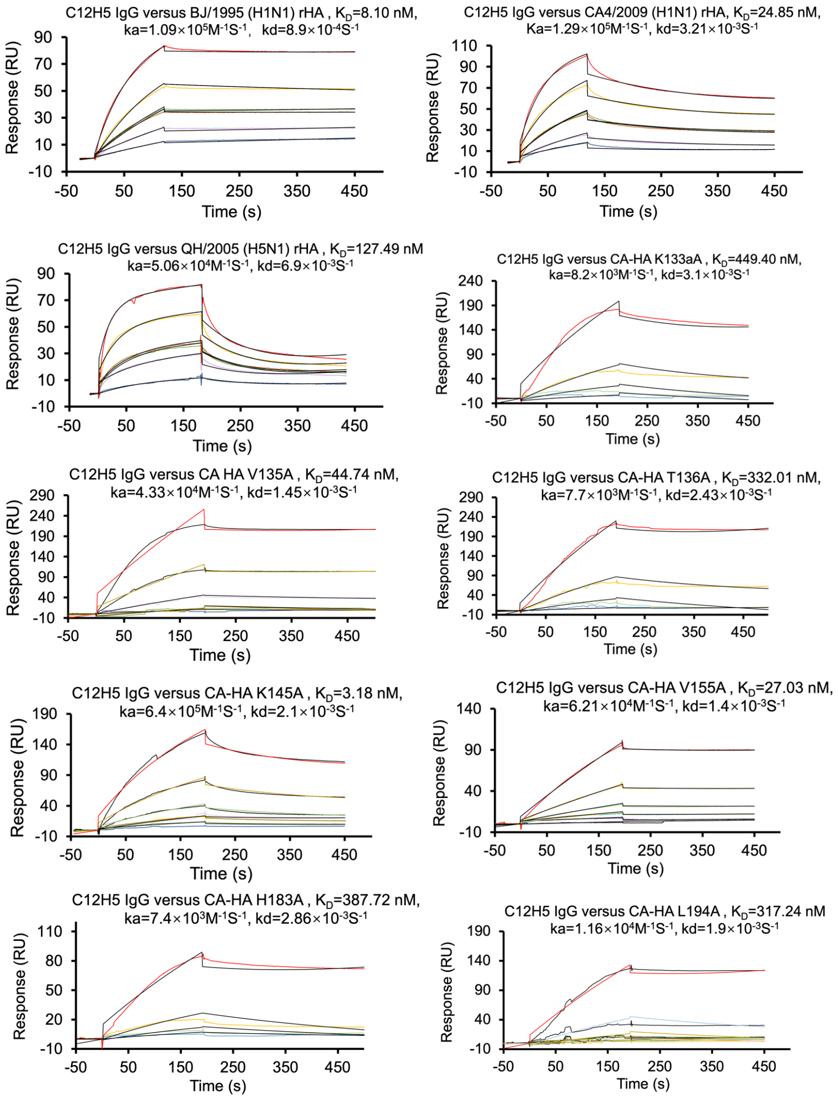

Supplementary Fig. 12. Binding curves of HA and MAb C12H5 in SPR. A total of 5 concentrations (160, 80, 40, 20, and 10 nM) of HA mutants (serial dilutions) were injected onto a MAb C12H5-bound chip. The kinetic constants between MAb C12H5 and HA/HA mutants are reported in Table S8. Coloured curves are the experimental traces obtained from surface plasmon resonance (SPR) experiments, and black curves indicate the best global fit for the data used to calculate the K_D values. Source data are provided as a Source Data file.

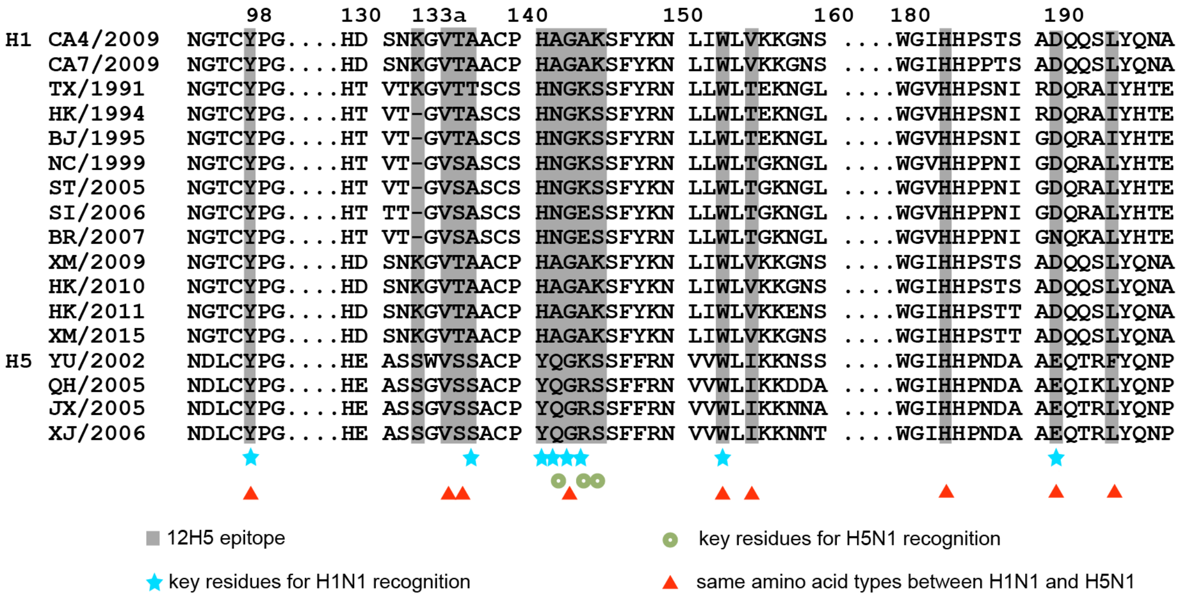

Supplementary Fig. 13. Sequence alignment of HA RBS of representative H1N1 and H5N1 isolates neutralized by mAb 12H5. A total of 15 sequences of the receptor binding site (RBS) domain (aa 94–198) of H1N1 and H5N1 strains neutralized by 12H5 were aligned to show their genetic conservation. The epitope residues of 12H5 are shaded in red. The critical residues for H1N1 recognition are indicated with an asterisk. The critical residues for H5N1 specific recognition are identified by a green circle. The same amino acid types between H1N1 and H5N1 are marked in red triangle.

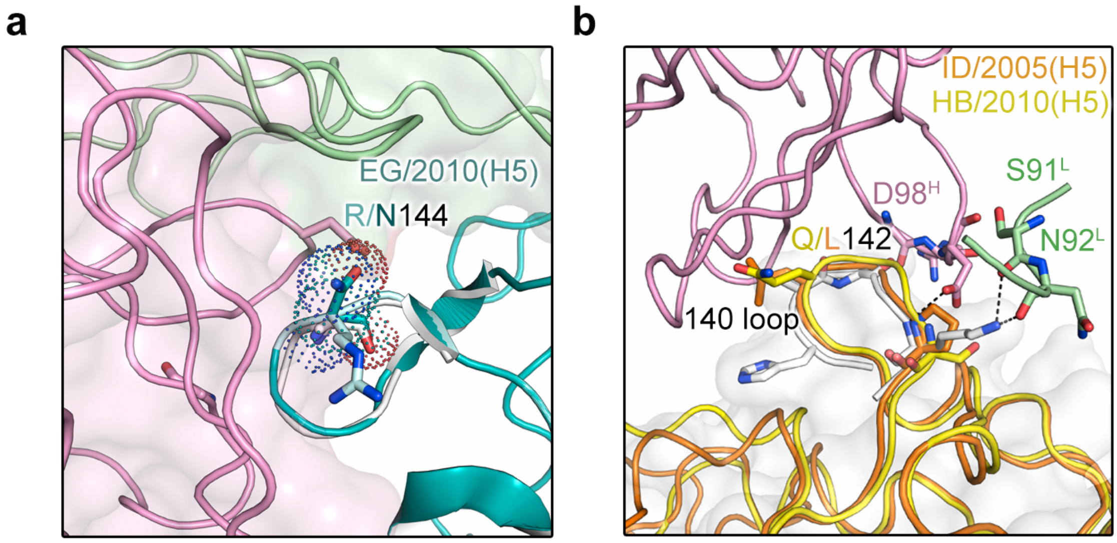

Supplementary Fig. 14. Structure analysis of key sites of 12H5 recognition to the H5N1 virus. a The structure of QH/2005 HA is unavailable, we used the structure of A/Duck/Egypt/10185SS/2010 HA (EG/2010, PDB code: 5E30) to analysis. R144N mutation on H5N1 EG/2010 HA might introduce glycans and obscure recognition by 12H5. **b** Q142 (A/Hubei/1/2010, HB/2010, PDB code: 4KTH) mutates to L142 (A/Indonesia/5/2005, ID/2005, PDB code: 4K62) might decrease the polarity and interfere with the close interaction of the 140- loop with 12H5. S145 (HB/2010) mutates to P145 (ID/2005) might decrease the flexibility of the 140- loop tip and cause a clash with 12H5.

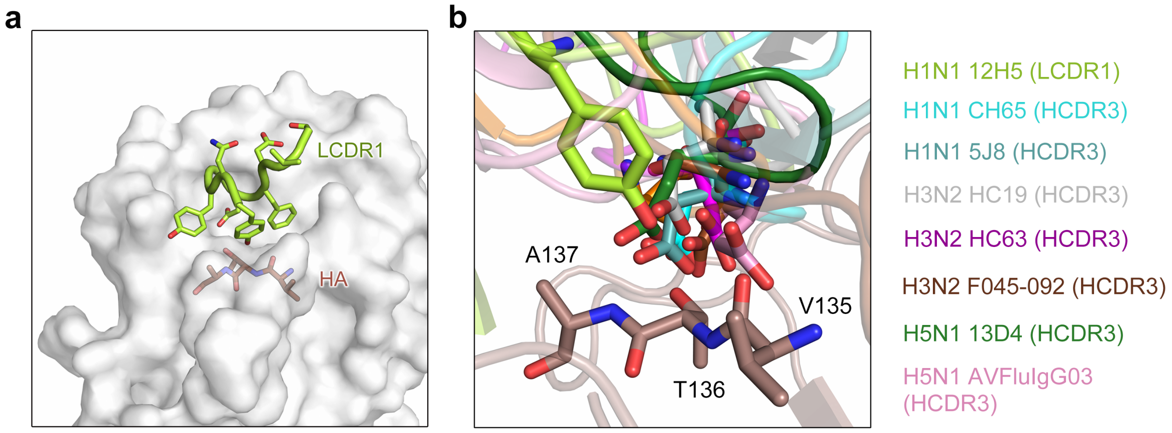

Supplementary Fig. 15. Overlay of the 12H5 LCDR tip with representative RBS-directed neutralizing antibodies. a Surface representation of the receptor binding site (RBS) domain in its interaction with LCDR1. **b** The Tyr at the tip of 12H5 LCDR3 (light green) is different from the Asp and Glu present in other antibodies. LCDR1, light chain complementarity determining region 1; HCDR3, heavy chain complementarity determining region 3. 12H5 (PDB code: 7FAH), CH65 (3SM5), 5J8 (4M5Z), HC19 (2VIR), HC63 (1KEN), Fo45-092 (4O5I), and AVFluIgG03 (5DUP).

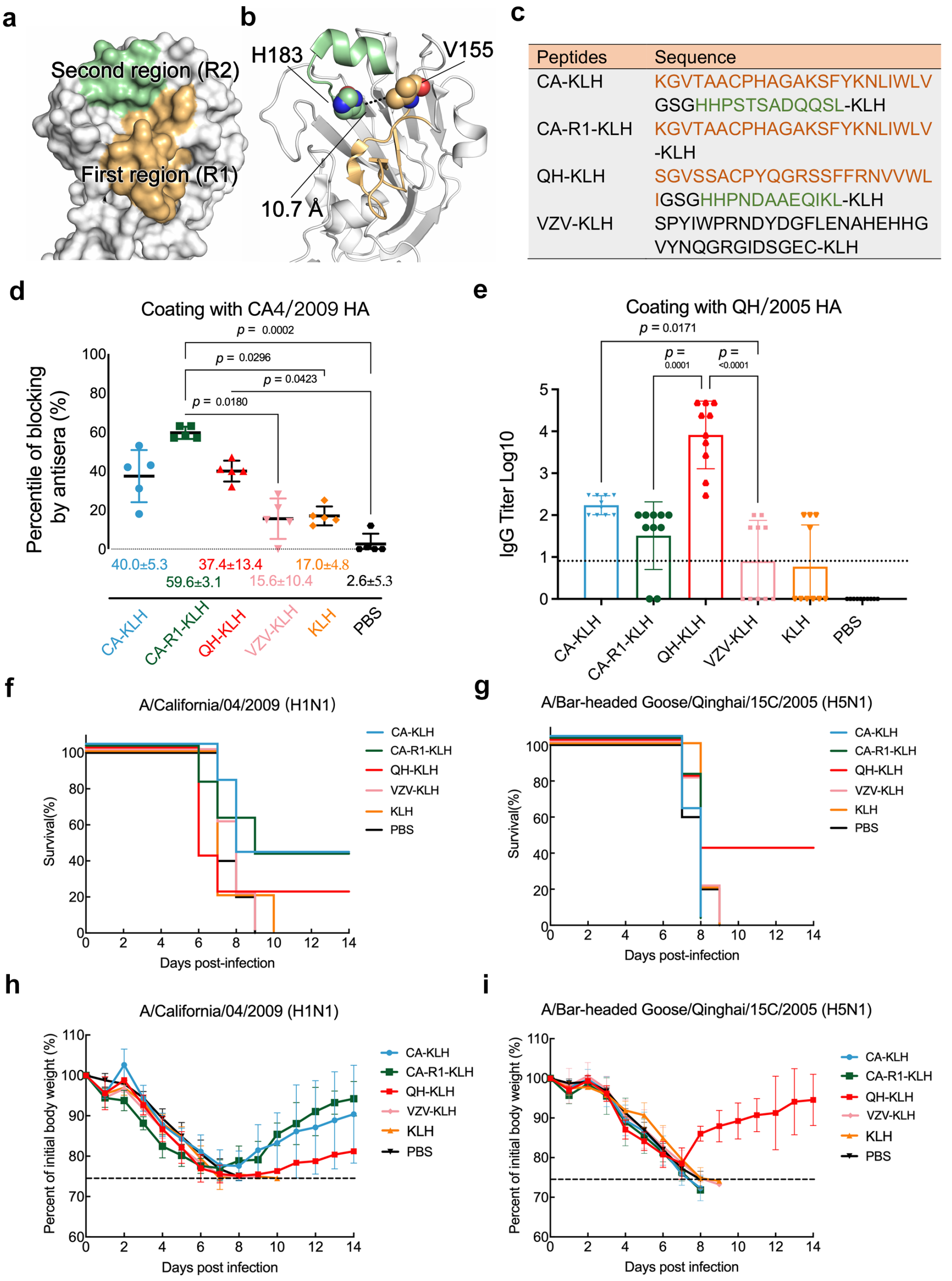

Supplementary Fig. 16. Rational design and immunogenicity assay of KLH-conjugated polypeptide based on the structure of C12H5 epitope. a and **b** Structure-based design of RBS peptide immunogens. The HA structure is shown in surface representation (**a**) and cartoon (**b**) with the 12H5 epitope highlighted in color. Region 1 (R1), in orange, runs from K133a to V155. R2, in blue, runs from H183 to L194. The spatial

distance between the two domains is about 10.7 Å, which is approximately equivalent to a length of a 3-aa peptide. We then designed a flexible GSG linker to join these two domains. **c** KLH-conjugated polypeptides: designation and sequence. **d** Binding of 12H5 to CA4/2009 HA is partly blocked by antisera of the KLH-conjugated polypeptides (n=5 per group, mean ± SD). **e** ELISA titer of antisera of KLH-conjugated polypeptides to QH/2005 HA (n=10 per group, mean ± SD). Statistical analyses in **d, e** was performed using Kruskal-Wallis with Dunn's multiple comparisons test (****$p<0.0001$; ***$p<0.001$; **$p<0.01$; *$p<0.05$). **f** to **i** Survival curves (**f, g**) and body weight monitoring (**h, i**) for BALB/c mice (n = 5 per group, mean ± SD) challenged with MA-CA4/2009 virus and MA-QH/2005 virus, respectively, after immunization with the KLH-conjugated polypeptides. Source data are provided as a Source Data file.

Supplementary Tables

Supplementary Table 1. Neutralization IC_{50} values of mouse antibody 12H5.

Subtypes	Strains	IC_{50}(μg/mL)	
		HI	MN
Seasonal H1N1	A/Texas/36/1991	1.10	0.07
	A/Hong Kong/134801/1994	0.20	0.55
	A/Beijing/262/1995	1.10	0.10
	A/New Caledonia/20/1999	0.25	0.55
	A/Shantou/104/2005	0.16	ND
	A/Solomon Is/3/2006	8.84	4.17
	A/Brisbane/59/2007	4.42	4.42
Pandemic H1N1	A/California/04/2009	4.42	8.84
	A/California/07/2009	4.42	8.84
	A/Xiamen/N514/2009	4.42	1.10
	A/Hong Kong/MB-1/2010	1.10	10.42
	A/Hong Kong/402618/2011	2.21	7.98
	A/Xiamen/s27/2015	ND	8.16
H5N1	A/Chicken/Hong Kong/YU22/2002	4.42	8.84
	A/Bar-headed Goose/Qinghai/15C/2005	4.42	8.84
	A/Migratory Duck/Jiangxi/2295/2005	1.99	ND
	A/Xinjiang/1/2006	3.56	10.06
H3N2	A/Brisbane/10/2007	>50	>50

* ND, not determined.

Supplementary Table 2. Influenza H1N1 and H5N1 viruses panel

Subtypes	Strains	Abbrev.	Vaccine candidate
Seasonal H1N1	A/Texas/36/1991	TX/1991	
	A/Hong Kong/134801/1994	HK/1994	
	A/Beijing/262/1995	BJ/1995	Yes
	A/New Caledonia/20/1999	NC/1999	Yes
	A/Solomon Is/3/2006	SI/2006	
	A/Brisbane/59/2007	BR/2007	Yes
Pandemic H1N1	A/California/04/2009	CA4/2009	
	A/California/07/2009	CA7/2009	Yes
	A/Xiamen/N514/2009	XM/2009	
	A/Hong Kong/MB-1/2010	HK/2010	
	A/Hong Kong/402618/2011	HK/2011	
	A/Xiamen/s27/2015	XM/2015	
H5N1	A/Bar-headed Goose/Qinghai/15C/2005	QH/2005	
	A/Migratory Duck/Jiangxi/2295/2005	JX/2005	
	A/Xinjiang/1/2006	XJ/2006	
B	B/Florida/4/2006	FL/2006	Yes

Supplementary Table 3. Data collection and refinement statistics for 12H5:HAhr^{CA} complex

	12H5:HAhr^{CA} (PDB: 7FAH)
Data collection	
Cell parameters (Å, º)	a=107.7, b=51.1, c=168.2
	α=90, β=106.9, γ=90
Space group	$P2_1$
Resolution range [a] (Å)	50.00-3.15 (3.20-3.15)
Wavelength (Å)	0.97930
Observed hkl (I>σ)	209,359
Unique hkl	30,642
Redundancy	11.8 (6.8)
Completeness (%)	99.8 (99.5)
Overall (I/σI)	19.2 (2.4)
R_{sym} [b] (%)	18.8 (92.6)
R_{pim} [c] (%)	7.7 (37.6)
CC1/2	0.994 (0.862)
Refinement	
Resolution range (Å)	38.61-3.15
Number of Reflections	30,631
R_{factor} [d]	26.6
R_{free} [e]	28.9
RMSD bond lengths (Å)	0.003
RMSD bond angles (°)	0.68
No. atoms	10,066
Protein	10,014
Glycan	52
Wilson B-factor (Å^2)	63
Average B-factors (Å^2)	107
Protein	108
HAhr^{CA}	51
12H5	136
Glycan	84
Ramachandran Plot	
Favoured and allowed region (%)	91.3
Generously allowed regions (%)	8.3
Disallowed regions (%)	0.4

[a] Numbers in parentheses refer to the highest resolution shell.
[b] $R_{sym} = \Sigma h\ \Sigma i|I_1(h) - <I(h)| / \Sigma h\ \Sigma i\ I_1(h)$
[c] $R_{pim} = \Sigma h SQRT(1/(N-1))\ \Sigma i|I(_1h) - <I(h)| / \Sigma h\ \Sigma i\ I(_1h)$
[d] $R_{factor} = \Sigma hkl||Fobs| - k|Fcalc||/\Sigma hkl|Fobs|$.
[e] R_{free} is calculated using the same equation as that for R factor but 5.0% of reflections were chosen randomly and omitted from the refinement

Supplementary Table 4. Cryo-EM data collection, refinement and validation statistics

	12H5: HA^{BJ} (PDB: 7YHK)
**Data collection and processing**	
Microscope	FEI TF30
Camera	Gatan K3
Magnification	91,000
Voltage (kV)	300
Electron exposure (e-/A2)	60
Defocus range (μm)	1.0-3.0
Pixel size (Å)	0.778
Micrographs (total)	4,059
Micrographs (used)	3,230
Total particle	828,505
Final particle images (no.)	137,484
Symmetry imposed	C3
Map resolution (Å)	3.14
FSC threshold	0.143
Map sharpening B factor (Å2)	-111.6
**Validation**	
MolProbity score	1.65
Clashscore	5.37
Poor rotamers (%)	0.76
RMS (bonds)	0.007
RMS (angles)	1.26
Ramachadran plot	
Favored (%)	94.80
Allowed (%)	5.03
Disallowed (%)	0.17

Supplementary Table 5. Interface identification and interaction analysis of 12H5:HAhr^{CA} by PISA Program

<table>
<tr><th colspan="4">Interface</th><th colspan="4">Interaction</th></tr>
<tr><th>HA</th><th>12H5</th><th>BSA(Å^2)[a]</th><th>Percentage[b]</th><th>Type[c]</th><th>12H5</th><th>Dist.(Å)</th><th>HA</th></tr>
<tr><td>**A:LYS63**</td><td>H chain</td><td>14.0</td><td>||</td><td>H</td><td>H:ASN52[ND2]</td><td>3.15</td><td>A:HIS141[O]</td></tr>
<tr><td>**A:GLU75**</td><td>H chain</td><td>28.2</td><td>||||</td><td>H</td><td>H:GLY33[N]</td><td>3.10</td><td>A:ALA142[O]</td></tr>
<tr><td>**A:GLU77**</td><td>H chain</td><td>30.7</td><td>||||</td><td>H</td><td>H:VAL96[N]</td><td>3.51</td><td>A:GLY143[O]</td></tr>
<tr><td>**A:LEU79**</td><td>H chain</td><td>4.5</td><td>|</td><td>H</td><td>H:ARG97[N]</td><td>3.66</td><td>A:GLY143[O]</td></tr>
<tr><td>**A:ALA137**</td><td>H chain</td><td>19.8</td><td>|||</td><td>H</td><td>H:ASP98[N]</td><td>3.05</td><td>A:GLY143[O]</td></tr>
<tr><td>**A:PRO140**</td><td>H chain</td><td>1.9</td><td>|</td><td>H</td><td>H:THR54[OG1]</td><td>3.57</td><td>A:HIS141[NE2]</td></tr>
<tr><td>**A:HIS141**</td><td>H chain</td><td>63.7</td><td>|||||||</td><td>H</td><td>H:ASP98[OD1]</td><td>2.87</td><td>A:LYS145[N]</td></tr>
<tr><td>**A:ALA142**</td><td>H chain</td><td>90.6</td><td>||||||||||</td><td>H</td><td>L:GLY30c[N]</td><td>3.52</td><td>A:ASP190[OD2]</td></tr>
<tr><td>**A:GLY143**</td><td>H chain</td><td>75.4</td><td>||||||||||</td><td>H</td><td>L:TYR32[OH]</td><td>3.74</td><td>A:VAL135[O]</td></tr>
<tr><td>**A:ALA144**</td><td>H chain</td><td>46.2</td><td>|||||||||</td><td>H</td><td>L:TYR32[OH]</td><td>3.68</td><td>A:THR136[OG1]</td></tr>
<tr><td>**A:LYS145**</td><td>H chain</td><td>21.7</td><td>||</td><td>H</td><td>L:ASP30b[OD2]</td><td>2.63</td><td>A:TYR98[OH]</td></tr>
<tr><td>**A:TYR98**</td><td>L chain</td><td>10.2</td><td>||||||||||</td><td>H</td><td>L:TYR32[OH]</td><td>3.23</td><td>A:ALA137[N]</td></tr>
<tr><td>**A:LYS133a**</td><td>L chain</td><td>63.6</td><td>|||||</td><td>H</td><td>L:SER91[O]</td><td>2.86</td><td>A:LYS145[NZ]</td></tr>
<tr><td>**A:GLY134**</td><td>L chain</td><td>0.8</td><td>|||</td><td>H</td><td>L:ASN92[O]</td><td>2.39</td><td>A:LYS145[NZ]</td></tr>
<tr><td>**A:VAL135**</td><td>L chain</td><td>12.7</td><td>|||</td><td>H</td><td>L:GLU93[OE1]</td><td>3.15</td><td>A:LYS133a[NZ]</td></tr>
<tr><td>**A:THR136**</td><td>L chain</td><td>25.1</td><td>|||||||||||</td><td>S</td><td>L:ASP30b[OD1]</td><td>3.60</td><td>A:HIS183[NE2]</td></tr>
<tr><td>**A:ALA137**</td><td>L chain</td><td>40.0</td><td>|||||</td><td>S</td><td>L:GLU93[OE1]</td><td>3.15</td><td>A:LYS133a[NZ]</td></tr>
<tr><td>**A:ALA144**</td><td>L chain</td><td>10.0</td><td>||</td><td></td><td></td><td></td><td></td></tr>
<tr><td>**A:LYS145**</td><td>L chain</td><td>82.2</td><td>|||||||</td><td></td><td></td><td></td><td></td></tr>
<tr><td>**A:TRP153**</td><td>L chain</td><td>16.2</td><td>|||||||||</td><td></td><td></td><td></td><td></td></tr>
<tr><td>**A:VAL155**</td><td>L chain</td><td>6.4</td><td>||||||||</td><td></td><td></td><td></td><td></td></tr>
<tr><td>**A:HIS183**</td><td>L chain</td><td>7.4</td><td>||||||||||</td><td></td><td></td><td></td><td></td></tr>
<tr><td>**A:PRO185**</td><td>L chain</td><td>0.5</td><td>||||</td><td></td><td></td><td></td><td></td></tr>
<tr><td>**A:ASP190**</td><td>L chain</td><td>36.5</td><td>|||||||</td><td></td><td></td><td></td><td></td></tr>
<tr><td>**A:LEU194**</td><td>L chain</td><td>37.2</td><td>|||||||||</td><td></td><td></td><td></td><td></td></tr>
<tr><td>**A:ASP225**</td><td>L chain</td><td>57.2</td><td>||||||</td><td></td><td></td><td></td><td></td></tr>
<tr><td>**A:GLN226**</td><td>L chain</td><td>24.1</td><td>|||||||||</td><td></td><td></td><td></td><td></td></tr>
<tr><td>**A:GLY228**</td><td>L chain</td><td>0.3</td><td>|</td><td></td><td></td><td></td><td></td></tr>
</table>

[a] BSA: Buried Surface Area; [b] ||||: Buried area percentage, one bar per 10%. [c] H, Hydrogen bond; S, Salt bridge.

Supplementary Table 6. Sequence identity of 12H5 epitopes in all H1N1 and H5N1 isolates

				Percent identity by subtype (%)	
Position	Consensus[a]	CA09/H1 sequence	QH05/H5 sequence	H1N1(8,907)[b]	H5N1(2,263)
98	Y (99.9)	Y	Y	Y (99.9)	Y (99.9)
133a	K/R (69.7)	K	S	K/R (87.6)	S (47.3), L (43.5)
135	V (95.5)	V	V	V (94.5)	V (99.7)
136	T (70.3)	T	S	T (88.6)	S (99.8)
137	A (81.3)	A	S	A (94.1)	S (69.5), A (30.5)
141	H (72.4)	H	Y	H (90.5)	Y (99.4)
142	A (77.9)	A	Q	A (77.4), N (12.3)	Q (80.0), L (13.7)
143	G (99.3)	G	G	G (99.5)	G (98.6)
144	A (58.5)	A	R	A (73.2), T (11.7)	R/K (50.1), N (22.2)
145	K (50.7)	K	S	K (63.4), N/S (18.6, 15.8)	S (72.1), P (27.9)
153	W (99.9)	W	W	W (99.9)	W (100)
155	V (72.6)	V	I	V (81.6)	I (99.1)
183	H 99.9)	H	H	H (99.5)	H (99.9)
190	D (73.2)	D	E	D (91.6)	E (99.7)
194	L (96.7)	L	L	L (97.1)	L (94.7)
Avg[c]	81.2			89.2	87.3

[a] Most common residue at position across all H1N1 and H5N1 sequences.

[b] Number of sequences available for subtype in the NCBI Influenza Database at the time of download on May 3, 2021.

[c] Average conservation of most common residue in subtype.

Supplementary Table 7. Comparison of escape mutant viruses in HAI assay using 12H5 MAbs

Virus Strains	IC_{50} (μg/mL)
**Wildtype virus**	
A/Hong Kong/134801/1994	0.20
A/New Caledonia/20/1999	0.25
A/Brisbane/59/2007	4.4
**Escape mutant**	
A/Hong Kong/134801/1994-G143R	> 50
A/New Caledonia/20/1999-K144T	> 50
A/Brisbane/59/2007-N142D	> 50
A/California/07/2009-A142E	> 50

HAI: hemagglutinin inhibition activity assay

Supplementary Table 8. Kinetic constants for binding activity of C12H5 to CA4/2009 HA mutants

HA	ka ($M^{-1}S^{-1}×10^4$)	kd ($s^{-1}×10^{-4}$)	K_D (nM)
Wildtype	12.90	32.05	24.85
Y98A	ND[a]	ND	ND
K133aA	0.82	31.10	449.40
V135A	4.33	14.50	44.74
T136A	0.77	24.30	332.01
A137E	ND	ND	ND
H141A	ND	ND	ND
A142E	ND	ND	ND
G143R	ND	ND	ND
A144E	ND	ND	ND
K145A	64.25	21.31	3.18
W153A	ND	ND	ND
V155A	6.21	14.10	27.03
H183A	0.74	28.65	387.72
D190A	ND	ND	ND
L194A	1.16	18.95	317.24

ka, *kd* and K_D are reported as the mean from two independent experiments.
[a] ND means the constant is not determinable.

Supplementary Table 9. 12H5 contact residues in H1N1 and H5N1 HAs.

Subtypes	Strains	98	133a	135	136	137	141	142	143	144	145	153	155	183	190	194
H1N1	CA4/2009	Y	K	V	T	A	H	A	G	A	K	W	V	H	D	L
	CA7/2009	Y	K	V	T	A	H	A	G	A	K	W	V	H	D	L
	TX/1991	Y	K	V	T	T	H	N	G	K	S	W	T	H	D	L
	HK/1994	Y	-	V	T	A	H	N	G	K	S	W	T	H	D	L
	BJ/1995	Y	-	V	T	A	H	N	G	K	S	W	T	H	D	L
	NC/1999	Y	-	V	S	A	H	N	G	K	S	W	T	H	N	L
	ST/2005	Y	-	V	S	A	H	N	G	K	S	W	T	H	D	L
	SI/2005	Y	-	V	S	A	H	N	G	E	S	W	T	H	D	L
	BR/2007	Y	-	V	S	A	H	N	G	E	S	W	T	H	N	L
	XM/2009	Y	K	V	T	A	H	A	G	A	K	W	V	H	D	L
	HK/2010	Y	K	V	T	A	H	A	G	A	K	W	V	H	D	L
	HK/2011	Y	K	V	T	A	H	A	G	A	K	W	V	H	D	L
	XM/2015	Y	K	V	T	A	H	A	G	A	K	W	V	H	D	L
H5N1	YU/2002	Y	S	V	S	S	Y	Q	G	K	S	W	I	H	E	F
	QH/2005	Y	S	V	S	S	Y	Q	G	R	S	W	I	H	E	L
	JX/2005	Y	S	V	S	S	Y	Q	G	R	S	W	I	H	E	L
	XJ/2006	Y	S	V	S	S	Y	Q	G	R	S	W	I	H	E	L
	EG/2010*	Y	-	V	S	S	Y	Q	G	R	S	W	T	H	E	L
	HB/2010*	Y	L	V	S	A	Y	Q	G	K	S	W	I	H	E	L
	ID/2005*	Y	S	V	S	S	Y	L	G	S	P	W	I	H	E	L

"-": denoted no residue at this position compared with A/California/04/2009 HA.
"*": Modeled H5 sequence in Supplementary Fig. 14.
